# Emergence of vancomycin-resistant enterococci in Switzerland: a nation-wide survey

**DOI:** 10.1186/s13756-019-0466-x

**Published:** 2019-01-17

**Authors:** Niccolò Buetti, Nasstasja Wassilew, Viktorija Rion, Laurence Senn, Céline Gardiol, Andreas Widmer, Jonas Marschall, for Swissnoso, C. Balmelli, M. C. Eisenring, S. Harbarth, S. P. Kuster, V. Masserey Spicher, D. Pittet, C. Ruef, H. Sax, M. Schlegel, A. Schweiger, N. Troillet, G. Zanetti

**Affiliations:** 1 Department of Infectious Diseases, Bern University Hospital, University of Bern, Freiburgstrasse, 3010 Bern, Switzerland; 2 Swissnoso, National Center for Infection Control, Bern, Switzerland; 3 UMR 1137 - IAME Team 5 - DeSCID: Decision SCiences in Infectious Diseases, control and care Inserm, University Paris Diderot, Sorbonne Paris Cité, Paris, France; 4 Service of Hospital Preventive Medicine, Lausanne University Hospital, Lausanne, Switzerland; 5 Swiss Federal Office of Public Health, Bern, Switzerland; 6 Division of Infectious Diseases & Hospital Epidemiology, University Hospital Basel, University of Basel, Basel, Switzerland

**Keywords:** Vancomycin-resistant enterococci, VRE, Outbreak, ST796, Hospital-acquired, Nosocomial

## Abstract

This nation-wide survey on the epidemiology of vancomycin-resistant enterococci (VRE) included 142 healthcare institutions and showed an increasing number of VRE colonizations and infections in Switzerland, probably for the most part due to nosocomial dissemination. The introduction and spread of a new clone, gaps in VRE screening policies as well as heterogeneity regarding the management of VRE clusters may be possible explanations.

**Electronic supplementary material:**

The online version of this article (10.1186/s13756-019-0466-x) contains supplementary material, which is available to authorized users.

## Introduction

Vancomycin-resistant enterococci (VRE) are multi-drug resistant organisms (MDROs) that can cause healthcare-associated infections and increase both length of stay and in-hospital mortality [1, 2]. The WHO listed VRE as a pathogen of high priority in its global list of important antibiotic-resistant bacteria [3]. In Europe, several countries reported an increasing proportion of vancomycin resistance among invasive isolates of *Enterococcus faecium* [4]. In Switzerland, VRE incidence is currently not being monitored for infection control purposes at a national level. Moreover, in recent years nosocomial VRE outbreaks have been reported from several hospitals in Switzerland [5–7], revealing that VRE is of concern to our healthcare system. Therefore, an update addressing all Swiss acute-care hospitals was deemed necessary to evaluate the current VRE epidemiology and identify possible gaps in the outbreak management strategies.

## Methods

**Fig. 1:**
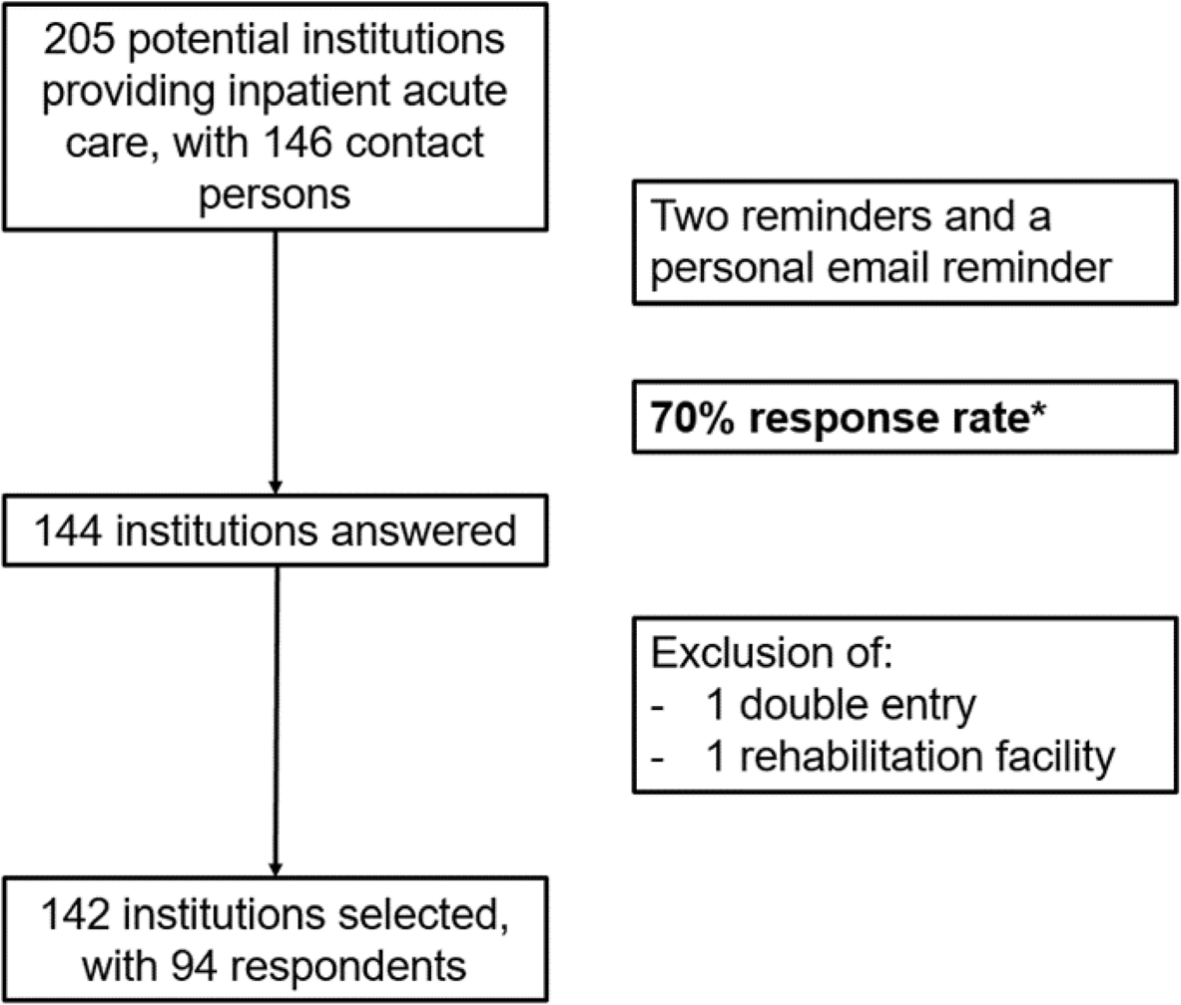
Survey institutions and respondents. Note: *Most of the 61 institutions that did not complete the survey were small hospitals (i.e., only three non-respondent hospitals had > 200 beds)

This survey included 205 public or private institutions providing inpatient care in Switzerland. The list of hospitals consisted of acute-care hospitals and was updated in March 2018 based on the official hospital list of the Federal Office of Public Health (http://www.bag.admin.ch) with inputs from the Swiss Hospital Society (http://www.hplus.ch). Psychiatric institutions, palliative care, long-term care facilities, rehabilitation facilities and pain therapy centers were excluded. Between May 1st and June 19th 2018, a 37-item questionnaire (Additional file 1) was sent via email to 146 contact individuals responsible for infection control at 205 acute-care institutions providing inpatient care (all hospitals and clinics with acute-care beds in Switzerland). Each email was sent in the respective local language (German, French or Italian). Two reminders as well as a personalized email were addressed to each non-responding institution. Overall, 144 institutions answered, corresponding to a 70% response rate. Most of the 61 institutions that did not complete the survey were small hospitals (i.e., only three non-responding hospitals had > 200 beds). After the exclusion of one double entry and one rehabilitation facility, 142 institutions were included in the final analysis (Fig. 1).

The survey was created, pre-tested locally and shared through the online platform SurveyMonkey®. We collected epidemiological data of VRE cases. Moreover, details on VRE outbreaks as well as information on VRE outbreak management strategies was inquired. An outbreak was defined as an unusual or unexpected increase in VRE colonizations and/or infections (i.e., ≥ 2 in the same time period in an individual hospital). A maximum of two major outbreaks per institution were analyzed in more detail.

All epidemiological information on both VRE in general and whether an institution had witnessed VRE outbreaks was institution-based (*n* = 142) and not respondent-based (*n* = 94) as certain contacts were responsible for multiple institutions.

Information about VRE outbreak management strategy was obtained from physicians who were involved in the management of a VRE outbreak and were therefore respondent-based (*n* = 14).

Data were extracted from the online platform to an Excel® spread-sheet, checked for accuracy and exported for descriptive analysis using SPSS (Version 25). The incidence rate was expressed in VRE cases per day.

## Results

### Responding institutions

Overall, 94 respondents answered for 142 institutions, accounting for 23′803 beds (out of 28′956 operated in Switzerland in 2018). Seventy-five percent (107) were small-size (< 200 beds), 18% (26) medium size (200–500 beds), and 6% (9) large-size institutions (> 500 beds). Ninety-two (65%) hospitals were located in the German-speaking part of Switzerland. There were 104 (73%) public hospitals among the 142 institutions.

### VRE epidemiology

From 1st January 2015 to 31st March 2018, VRE cases were observed in one third of hospitals (46/142, 32%). Overall, 652 VRE patients were reported, of which 67 (10%) represented invasive infections.

**Fig. 2:**
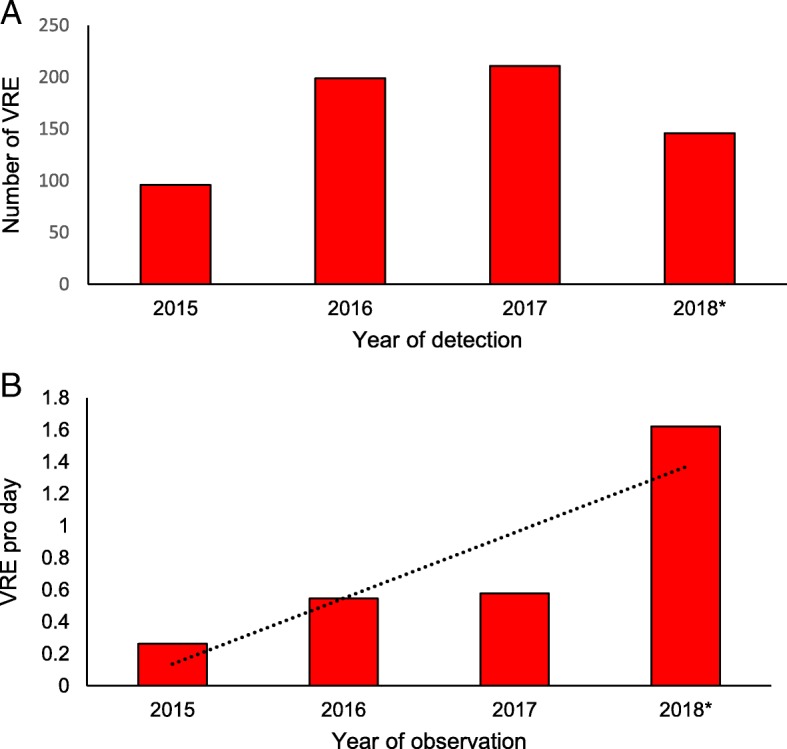
Number of VRE cases detected (**a**) and VRE incidence per day (**b**) from 2015 to 2018 in 142 Swiss institutions. Note: Fig. B with trend line (dotted). VRE: vancomycin resistant enterococci. *in 2018 only data for the first quarter were included

The total number of VRE cases increased from 96 in the year 2015 to 146 in the first three months of 2018 (Fig. 2a). Of note, the incidence rate increased from 0.26 cases/day in 2015 to 1.58 cases/day in 2018 in the approximately > 23′000 beds observed (Fig. 2b).

### VRE outbreaks in Switzerland

Twenty-three outbreaks were reported during the study period. Among the 20 major outbreaks analyzed, 250 VRE cases including 10 bacteremias (4%) were observed. Eight outbreaks took place on internal medicine floors, five in a surgical unit and four in an intensive care unit. Nine, five and two outbreaks showed a *vanB*, *vanA* and mixed pattern of *vanA* and *vanB* resistance types, respectively.

**Fig. 3:**
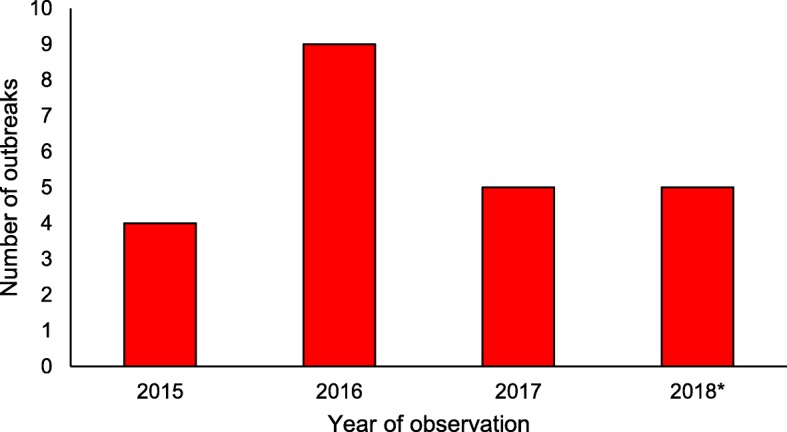
VRE outbreaks in Switzerland per year from 1st January 2015 to early April 2018. Note:* in 2018 only data until the beginning of April were included

The mean number of outbreaks per year was seven; in 2016 nine outbreaks were observed (Fig. 3). From 1st January 2018 to the beginning of April 2018 five outbreaks were observed, four of which were ongoing (all of them located in the German-speaking part of Switzerland) when the data collection ended. Seventy percent (102/146) of new VRE cases in 2018 were outbreak-related (i.e., hospital-acquired).

### VRE outbreak management strategies

Frequently used VRE outbreak management strategies were contact precautions for VRE colonized or infected patients (14 of 14 respondents, 100%), contact tracing (13/14, 93%) and pre-emptive contact precautions for high-risk contact patients (12/14, 86%) until negative screening samples were collected, reinforcement of hand hygiene compliance (14/14, 100%), and implementation of disinfectant based environmental cleaning (12/14, 86%). Screening samples of healthcare workers (2/14, 14%) as well as decolonization of VRE patients (1/14, 7%) were infrequently used measures. Heterogeneity was noted regarding the following measures: temporary ward closure (implemented in 8/14, 57%) or temporary re-organization of wards into sectors (7/14, 50%), cohorting of contact patients (5/14, 36%), staff cohorting (7/14, 50%), active screening cultures irrespective of exposition (performed in 10/14, 71%), and environmental screening cultures (only performed in 5/14, 36%). Only two hospitals implemented antimicrobial stewardship measures during an outbreak.

### Emergence of a new clone: The experience of Bern University hospital

In early 2018, the emergent clone VRE ST796 was detected for the first time and then found to produce multiple secondary clusters at Bern University Hospital, as previously reported [8]. This outbreak was ongoing when the survey was closed. VRE colonization was encountered in 68 patients, of whom 56 (86%) were affected by the ST796 clone. Five patients developed an invasive infection with this clone. Before this outbreak, ST796 had exclusively been described in Australia and New Zealand where it exhibited high transmissibility [9]. In the core gene multilocus sequence typing (cgMLST), all ST796 isolates were found to be virtually indistinguishable, underlining the epidemiologic linkage among these cases [8].

## Discussion

This nationwide survey on the VRE epidemiology is representative of Switzerland by including 144 institutions and characterized by an excellent response rate of 70%. The survey revealed an increasing number of VRE cases detected in 2018, which correlates with an increased number of outbreaks observed in the German-speaking part of Switzerland during the first three months of 2018.

This trend is in line with the rise of VRE prevalence and VRE outbreaks reported in some of the surrounding European countries, notably Italy and Germany, as reported by the European Center for Disease Prevention and Control [10, 11]. Interestingly, the European map is very heterogeneous concerning the prevalence of VRE and follows no distinct geographical pattern compared to many other multi-resistant bacteria. For example, France and Austria experienced a comparatively lower level of reported invasive isolates. To date, the reasons for the different VRE distribution in Europe remain unknown.

Recently, the efficient dissemination of a new clone (ST796) was described in two hospitals that participated in this survey [8]. The clinical significance of this strain compared to other VRE strains remains to be determined. A high rate of bloodstream infections with VRE ST796 amongst all sequence typed *E. faecium* bacteremias was observed in Australia, where ST796 was first described [9, 12]. However, the VRE prevalence is higher in Australia than in Switzerland and the rate of invasive infections probably reflect the high colonization prevalence in the patient population. Certainly, this clone has been characterized by a rapid intra- and inter-hospital spread with a propensity to adapt, probably in response to specific hospital environments [9, 13]. Moreover, a recent Swiss survey of screening practices for detecting carriers of MDROs illustrated a lack of awareness of the potential spread of VRE by means of unidentified carriers (manuscript in preparation, personal communication, S. Harbarth, Geneva). The marked upward trend in incidence is of particular concern, as several outbreaks were still ongoing in early April 2018. Moreover, a heterogeneity regarding the management of VRE outbreaks appears to characterize current infection prevention and control practices in Switzerland.

This study has several limitations. First, mean incidence rates were calculated using days as a denominator, leading to possible overestimation of the total incidence (e.g., seasonal increase of VRE cases during a specific year of observation). Second, an external validation of the respondents’ answers was not performed. Third, bacteremia and invasive infection rates should be interpreted with caution as these data were not available in all included institutions. Fourth, we excluded long-term care facilities and rehabilitation centers, which may represent an underestimated reservoir of multi-drug resistant organisms [14]. Fifth, our outbreak definition included both small clusters and large outbreaks; however, only 30% of the observed outbreaks included < 5 VRE detections. Finally, we cannot rule out the possibility that a patient with VRE carriage was recorded by more than one institution due to multiple presentations, leading to a possible overestimation of the total burden of VRE.

In conclusion, these findings highlight the emergence of VRE in parts of Switzerland not affected before, probably for the most part in the nosocomial setting. A harmonized nationwide strategy for VRE containment that includes active screening surveillance, uniform standards of detection and outbreak management, reporting at a national level with a central surveillance as well as guidance for patient transfers should therefore be implemented.

## Additional file

Additional file 1: English version of the questionnaire (translated from German, French and Italian). (PDF 563 kb)

## Abbreviations

MDRO: Multi-drug resistant organism
VRE: Vancomycin-resistant enterococci
