# Supplementary material for: Emergence of vancomycin-resistant enterococci in Switzerland: a nation-wide survey
Source: Antimicrob Resist Infect Control. 2019 Jan 17;8:16. doi: 10.1186/s13756-019-0466-x (PMC6337856; doi:10.1186/s13756-019-0466-x)
Supplement: Supplementary file 1 — English version of the questionnaire (translated from German, French and Italian). (PDF 563 kb) [file 13756_2019_466_MOESM1_ESM.pdf]

VRE EPIDEMIOLOGY AND OUTBREAKS IN SWITZERLAND – QUERY BY SWISS HOSPITALS

Introduction

In the years 2016-2018 there have been a few outbreaks with vancomycin-resistant enterococci (VRE) in Switzerland. Although multiple other Swiss centers have seen clusters or even outbreaks with VRE in the past, the scope of the problem is currently unknown. Support for understanding the Swiss VRE epidemiology also comes from ANRESIS, the Swiss Center for Antimicrobial Resistance, but is not real-time due to data processing requirements. On behalf of Swissnoso, National Center for Infection Control, we would like to collect information on cases your center has seen to obtain a timely picture of the VRE epidemiology in Switzerland.

Directions:

- This questionnaire should be filled out by a person knowledgeable about infection control practices in your facility. Please do not leave any questions unanswered.

- This survey will take about 10 min of your worktime in case you observed VRE outbreak in your facility in the last three years and is of utmost importance to understand the VRE epidemiology in Switzerland.

- In case you did not observe VRE outbreak in your facility in the last three years, this survey will take no more than 2 min of your worktime.

Your responses to this survey will be confidential. Your participation in this survey is crucial to the success of the project! Thank you very much for your effort!

If you have any questions or need assistance in completing this questionnaire, please call:

(+41)XX XXX XX XX

Monday through Friday

9:00 am – 4:00 pm

Abbreviations & Definitions:

VRE: Vancomycin resistant Enterococci (with Amoxicillin Resistance too)

ICU: Intensive Care Unit

Outbreak: Settings where there is an unusual or unexpected increase of cases (≥ 2 in the same time period on the same ward) of infections due to VRE already isolated in the hospital with or without molecular analysis of strains

PERSONAL DETAILS AND HOSPITAL DEMOGRAPHICS

1. Your details

   1.1. Name of your hospital/ clinic

1.2. Address of your hospital/ clinic

1.3. Name of the person completing this survey

1.4. Your position

1.5. Your department

1.6. Your email address

2. What is the number of acute care beds in your hospital?

VRE EPIDEMIOLOGY:

3. Have you seen VRE cases (either colonized or infected patients) in the years 2015, 2016, 2017 and/or 2018?

    1.1. Yes
    1.2. No (If No -> direct to: Thank you very much for your participation in this survey)

4. If yes, can you list the number of cases for each of these calendar years?

2015 (from 1st January to 31st December)
Total number
Number of Infections (if known):
BSI:
Others:

2016 (from 1st January to 31st December)
Total number
Number of Infections (if known)
BSI:
Others:

2017 (from 1st January to 31st December)
Total number
Number of Infections (if known)
BSI:
Others:

2018 (from 1st January to 31st March 2018)
Total number
Number of Infections (if known)
BSI:
Others:

VRE OUTBREAK DATA:

5. Did you **ever** observe an outbreak (for definition see above) with VRE in your institution?
   Yes
   No (If No -> direct to: Thank you very much for your participation in this survey)

6. If yes, did you observe more than one outbreak?
   Yes
   No (If No -> direct to: Outbreak description)

7. If yes, how many outbreaks did you observe in 2015-2018?
   Total number of outbreaks in 1/2015-4/2018

OUTBREAK DESCRPTION:

(please describe max. 2 biggest outbreaks)

	1st Outbreak	2nd Outbreak
1. When did the outbreak occur?	From:<br>To:	From:<br>To:
2. Where (which unit/department)?	2.1. Intensive care unit (ICU)<br>2.2. Surgery unit<br>2.3. Hemato-oncology unit<br>2.4. Internal medicine unit<br>2.5. Other: …	2.1. Intensive care unit (ICU)<br>2.2. Surgery unit<br>2.3. Hemato-oncology unit<br>2.4. Internal medicine unit<br>2.5. Other: …
3. Was the first case imported from another hospital?	a. Yes<br>b. No<br>c. Unknown<br><br>If yes, from a foreign country hospital?<br>a. Yes<br>b. No<br>c. Unknown	d. Yes<br>e. No<br>f. Unknown<br><br>If yes, from a foreign country hospital?<br>d. Yes<br>e. No<br>a. Unknown
4. How many outbreak-related colonizations/ infections did you identify?	4.1. Total number:<br><br>Of those:<br>4.2. Colonizations: Number<br>4.3. Invasive infections: Number<br><br>If invasive infections, of those (number):<br>a. Bloodstream infection:<br>b. Urinary infections:<br>c. Intra-abdominal sample<br>d. Other (e.g., respiratory etc)	4.1. Total number:<br><br>Of those:<br>4.2. Colonizations: Number<br>4.3. Invasive infections: Number<br><br>If invasive infections, of those (number):<br>a. Bloodstream infection:<br>b. Urine infections:<br>c. Intra-abdominal sample<br>d. Other (e.g., respiratory etc)
5. Did you perform molecular confirmation?	a. Yes<br>b. No<br><br>If yes, which subtype has been identified?<br><br>a. Van A<br>b. Van B	a. Yes<br>b. No<br><br>If yes, which subtype has been identified?<br><br>a. Van A<br>b. Van B

	c. Van A and Van B	c. Van A and Van B
6. Did you perform molecular typing of VRE strains?	a. Yes<br>b. No<br><br>If yes, was the outbreak with the same strain:<br>a. Yes<br>b. No<br><br>If with the same strain, are you willing to send the VRE stains of this outbreak for typing?<br>a. Yes<br>b. No<br><br>In the future, in case of a new outbreak in your hospital, would you agree to send your strains for whole genome sequencing in the context of a national surveillance?<br>a. Yes, but only if all costs are covered<br>b. Yes, but only if costs are at least partially covered<br>c. Yes, we would sequence for whole genome even if we have to cover all costs<br>d. No	a. Yes<br>b. No<br><br>If yes, was the outbreak with the same strain:<br>c. Yes<br>d. No<br><br>If with the same strain, are you willing to send the VRE stains of this outbreak for typing?<br>e. Yes<br>f. No<br><br>In the future, in case of a new outbreak in your hospital, would you agree to send your strains for whole genome sequencing in the context of a national surveillance?<br>a. Yes, but only if all costs are covered<br>b. Yes, but only if costs are at least partially covered<br>c. Yes, we would sequence for whole genome even if we have to cover all costs<br>d. No
7. Notification: Have you notified someone outside your institution of this outbreak?	7.1. Federal Office of Public Health<br>a. Yes<br>b. No<br>7.2. Cantonal physician<br>a. Yes<br>b. No<br>7.3. Other entities<br>a. Yes -> Which?<br>b. No	7.1. Federal Office of Public Health<br>a. Yes<br>b. No<br>7.2. Cantonal physician<br>a. Yes<br>b. No<br>7.3. Other entities<br>a. Yes -> Which?<br>b. No
8. Management: Have you	8.1. .. implemented contact precautions for VRE cases?<br>a. Yes<br>b. No<br>8.2. … implemented contact precautions for patients exposed to VRE cases (“contacts”)?<br>a. Yes<br>b. No<br>8.3. … enforced hand hygiene?<br>a. Yes<br>b. No	8.1. .. implemented contact precautions for VRE cases?<br>a. Yes<br>b. No<br>8.2. … implemented contact precautions for patients exposed to VRE cases (“contacts”)?<br>a. Yes<br>b. No<br>8.3. … enforced hand hygiene?<br>a. Yes<br>b. No

	8.4. … stopped patient admissions temporarily?<br>a. Yes<br>b. No<br>8.5. … created different sectors for VRE-positive patients?<br>a. Yes<br>b. No<br>High-risk contacts?<br>a. Yes<br>b. No<br>8.6. … cohorted staff?<br>a. Yes<br>b. No<br>8.7. … enhanced environmental cleaning?<br>a. Yes<br>b. No<br>8.8. … marked the contact patients with a special alert system?<br>a. Yes<br>b. No<br>8.9. … screened contact patients?<br>a. Yes<br>b. No<br>8.10. … performed routine screening cultures in specific units (irrespective of exposition)?<br>a. Yes<br>b. No<br>8.11. … implemented an antimicrobial stewardship strategy?<br>a. Yes<br>b. No<br>8.12. … screened health care workers (HCW)?<br>a. Yes<br>b. No<br>8.13. … obtained screening samples from the environment?<br>a. Yes<br>b. No<br>8.14. … attempted to decolonize VRE-positive patients and/or contacts?<br>a. Yes<br>b. No	8.4. … stopped patient admissions temporarily?<br>a. Yes<br>b. No<br>8.5. … created different sectors for for VRE-positive patients?<br>c. Yes<br>d. No<br>High-risk contacts?<br>c. Yes<br>d. No<br>8.6. … cohorted staff?<br>a. Yes<br>b. No<br>8.7. enhanced environmental cleaning?<br>a. Yes<br>b. No<br>8.8. … marked the contact patients with a special “alert-system”?<br>a. Yes<br>b. No<br>8.9. …screened contact patients?<br>a. Yes<br>b. No<br>8.10. … performed routine screening cultures in specific units (irrespective of exposition)?<br>a. Yes<br>b. No<br>8.11. … implemented an antimicrobial stewardship strategy?<br>a. Yes<br>b. No<br>8.12. … screened Health Care Workers (HCW)?<br>a. Yes<br>b. No<br>8.13. … obtained screening samples from the environment?<br>a. Yes<br>b. No<br>8.14. … attempted to decolonize VRE-positive patients and/or contacts?<br>a. Yes<br>b. No
